# Perceptions of individuals living with spinal cord injury toward preference-based quality of life instruments: a qualitative exploration

**DOI:** 10.1186/1477-7525-12-50

**Published:** 2014-04-14

**Authors:** David GT Whitehurst, Nitya Suryaprakash, Lidia Engel, Nicole Mittmann, Vanessa K Noonan, Marcel FS Dvorak, Stirling Bryan

**Affiliations:** 1 Faculty of Health Sciences, Simon Fraser University, Burnaby, British Columbia, Canada; 2 International Collaboration on Repair Discoveries (ICORD), Vancouver, British Columbia, Canada; 3 Centre for Clinical Epidemiology and Evaluation, Vancouver Coastal Health Research Institute, Vancouver, British Columbia, Canada; 4 Health Outcomes and Pharmaco Economics (HOPE) Research Centre, Sunnybrook Health Sciences Centre, Toronto, Ontario, Canada; 5 Department of Pharmacology, University of Toronto, Toronto, Ontario, Canada; 6 The International Centre for Health Innovation, Richard Ivey School of Business, University of Western Ontario, London, Ontario, Canada; 7 Rick Hansen Institute, Vancouver, British Columbia, Canada; 8 Division of Spine, Department of Orthopaedics, University of British Columbia, Vancouver, British Columbia, Canada; 9 School of Population and Public Health, University of British Columbia, Vancouver, British Columbia, Canada

**Keywords:** Preference-based outcomes, Health-related quality of life, Quality-adjusted life years, Spinal cord injury, Disability, Qualitative research

## Abstract

**Background:**

Generic preference-based health-related quality of life instruments are widely used to measure health benefit within economic evaluation. The availability of multiple instruments raises questions about their relative merits and recent studies have highlighted the paucity of evidence regarding measurement properties in the context of spinal cord injury (SCI). This qualitative study explores the views of individuals living with SCI towards six established instruments with the objective of identifying ‘preferred’ outcome measures (from the perspective of the study participants).

**Methods:**

Individuals living with SCI were invited to participate in one of three focus groups. Eligible participants were identified from Vancouver General Hospital’s Spine Program database; purposive sampling was used to ensure representation of different demographics and injury characteristics. Perceptions and opinions were solicited on the following questionnaires: 15D, Assessment of Quality of Life 8-dimension (AQoL-8D), EQ-5D-5L, Health Utilities Index (HUI), Quality of Well-Being Scale Self-Administered (QWB-SA), and the SF-36v2. Framework analysis was used to analyse the qualitative information gathered during discussion. Strengths and limitations of each questionnaire were thematically identified and managed using NVivo 9 software.

**Results:**

Major emergent themes were (i) general perceptions, (ii) comprehensiveness, (iii) content, (iv) wording and (v) features. Two sub-themes pertinent to content were also identified; ‘questions’ and ‘options’. All focus group participants (n = 15) perceived the AQoL-8D to be the most relevant instrument to administer within the SCI population. This measure was considered to be comprehensive, with relevant content (i.e. wheelchair inclusive) and applicable items. Participants had mixed perceptions about the other questionnaires, albeit to varying degrees.

**Conclusions:**

Despite a strong theoretical underpinning, the AQoL-8D (and other AQoL instruments) is infrequently used outside its country of origin (Australia). Empirical comparative analyses of the favoured instruments identified in this qualitative study are necessary within the context of spinal cord injury.

## Background

Measurements of health provide a focal point for many claims for evidence-based reform, irrespective of whether such measures are used to monitor outcomes of care within a specific clinical population, to diagnose illness, or to study the output of a health system as a whole. Selecting an appropriate health measure for a particular task is not straightforward, often due to the plethora of available outcome measures that purport to measure the same (or similar) concept. Outcome measurement for the purposes of economic evaluation is one such scenario. Increasing support for economic evaluation within a cost-utility framework, where health benefits are quantified using the quality-adjusted life year (QALY) metric, has ensured that preference-based health-related quality of life (HRQoL) instruments have been the focus of extensive psychometric evaluation across a broad range of clinical conditions and community-based samples. Spinal cord injury (SCI) is one clinical area that has not received this level of attention from the outcomes measurement or health economics research communities [1].

Valuations for health states can be measured directly from individuals using preference elicitation techniques such as standard gamble or time trade-off, or indirectly through the use of preference-based HRQoL instruments [2]. The latter approach is widely-used due to the availability of general population-based, ‘off the shelf’ values for every possible combination of responses for a particular instrument. The popularity of indirect measurement is not merely a reflection of the relative ease of data collection in comparison to time-consuming and expensive direct elicitation methods. There are theoretical and normative arguments for using general population values as opposed to patient values [3-5], and national guidelines in multiple jurisdictions require health benefits to be valued in a manner that reflects societal preferences [6-8].

Preference-based HRQoL instruments are, typically, generic outcome measures, meaning that they permit comparisons across disease areas. Preference-based instruments are made up of two constituent parts. The ‘descriptive system’ – i.e. the questions and respective response options that comprise the measure – defines respondents’ HRQoL as one of a finite number of health states. The ‘valuation system’ provides the scores that are attached to each health state (often referred to as health state valuations or utility scores/weights), which represent the relative value that society places on living in each health state. These scores fall on a scale where one indicates full health and zero represents a health state equivalent to death. Within the context of SCI, further details about the merits of preference-based HRQoL measurement, including a summary of the differences that exist between alternative generic instruments, is available elsewhere [9]. A recent systematic review that explored the use of preference-based HRQoL measures in SCI research concluded that there was limited conceptual or empirical research from which to justify the use of any of the available instruments [9]. Accordingly, the primary objective of the current study was to explore the perceptions of individuals living with SCI toward the different generic preference-based HRQoL measures that are regularly used in applied research. More specifically, the study examines the extent to which different measures enable individuals living with SCI to *describe* their health state in a manner deemed appropriate by respondents themselves. Although the research question focuses exclusively on preference-based instruments and individuals with SCI, it was anticipated that our approach would also provide insight regarding respondents’ general thoughts about the concept of standardized outcome measurement.

## Methods

### Design & sample

A qualitative study design was used, consisting of focus group discussions with individuals living with SCI. This approach was chosen in order to capture multiple perspectives in an engaging, interactive group setting. Purposive sampling was employed to ensure a range of participant and injury-related characteristics were represented in the sessions: these characteristics were gender, type of injury (i.e. non-traumatic SCI and traumatic SCI), time since injury, and severity of injury as reflected in the anatomical level. Individuals were eligible to take part in a focus group if they (i) were an adult aged 19 years or older, (ii) had a spinal cord injury (irrespective of the cause of injury or level of completeness, with the exception of non-traumatic SCI caused by metastatic disease), (iii) resided in the Greater Vancouver area, and (iv) were able to converse in English. Exclusions were a diagnosis with severe cognitive impairment not amenable to treatment, residence in a long term care facility, and/or ongoing medical or surgical complications requiring hospitalization.

### Recruitment & consent procedure

Potential participants were identified from records held at Vancouver General Hospital’s Spine Program (British Columbia, Canada); records were screened by the Spine Program staff and a list was generated. Each person on this list was mailed a package containing a letter of invitation and a consent form. The letter of invitation explained that the focus group discussion would last no longer than two hours and that participants would be compensated for their time at the end of the session. If an individual wanted to participate, they were required to sign and return the consent form (in a prepaid, preaddressed envelope provided in the package) and await further contact by the study coordinator. Two weeks after the invitation letter mailout, a follow-up phone call was made by Program staff to ascertain whether invitees were interested in participating in the study. Individuals’ consent to participate was communicated to the study coordinator. All consenting participants were sent a subsequent package comprising a confirmation letter with details of the location and time of the focus group, and copies of the six preference-based HRQoL questionnaires described in the following section. Participants were asked to review the questionnaires prior to attending the focus group; there was no explicit request for participants to complete any of the questionnaires.

### Outcome measures/instruments

Instrument selection for this study was directed, in part, by the results of a systematic review of preference-based HRQoL measurement in the context of SCI [9]; it was not feasible to include all available instruments (and variants of instruments) and, therefore, choices had to be made (see ‘Strengths and Limitations’ section). The major finding of the systematic review was the absence of supportive psychometric evidence for any current preference-based HRQoL instrument. Accordingly, despite the fact that there is considerable variation in the prevalence of different measures in the economic evaluation literature, the descriptive systems of six preference-based HRQoL instruments were selected to ensure comprehensive consideration of available options: 15D [10], Assessment of Quality of Life 8-dimension (AQoL-8D) [11], EQ-5D-5L [12], Health Utilities Index [13], Quality of Well-Being Scale Self-Administered (QWB-SA) [14], and the SF-6D (SF-36v2) [15]. Table 1 provides a summary of the descriptive systems for each of these outcome measures.

**Table 1:** **Key properties of the six preference-based HRQoL instruments discussed by focus group participants**^**a**^

**Instrument**	**Dimensions/domains within the descriptive system**	**Number of items used to derive a health state valuation**	**Number of unique health states**^**b**^
15D [10]	15: mobility, vision, hearing, breathing, sleeping, eating, speech, elimination, usual activities, mental function, discomfort and symptoms, depression, distress, vitality, sexual activity	15 questions, each with 5 levels of response	More than 30 billion
AQoL-8D [11]	8: independent living, senses, pain, mental health, happiness, self worth, coping, relationships	35 questions, with between 4 and 6 levels of response	More than 60 trillion
EQ-5D-5L [12]	5: mobility, self-care, usual activities, pain/discomfort, anxiety/depression	5 questions, each with 5 levels of response	3125
HUI [13]	6: sensation, mobility, emotion, cognition, self-care, pain	15 questions, with between 4 and 6 levels of response^c^	8,000
QWB-SA [14]	5: acute and/or chronic symptoms, self-care, mobility, physical functioning, performance of usual activity	At least 71 questions, with varying response formats^d^	1,215
SF-6D (SF-36v2) [15]	6: physical functioning, role limitations, social functioning, pain, mental health, vitality	11 (of 36) questions, with between 3 and 6 levels of response^e^	18,000

^a^All preference-based HRQoL measures provide scores that are interpretable on a 0–1 scale, where 0 = health state equivalent to death and 1 = full health. Discussion sequences were as follows: HUI, EQ-5D-5L, 15D, AQoL-8D, QWB-SA, SF-36v2 (focus group 1); 15D, AQoL-8D, QWB-SA, SF-36v2, HUI, EQ-5D-5L (focus group 2); QWB-SA, SF-36v2, HUI, EQ-5D-5L, 15D, AQoL-8D (focus group 3).

^b^‘Number of unique health states’ refers to the total number of different ways to complete the instrument. For example, for the EQ-5D-5L there are 5^5^ possible response combinations (5x5x5x5x5 = 3125).

^c^Although there are 15 items contained in the descriptive system of the Health Utilities Index, scoring algorithms for the HUI Mark 2 (HUI2) and HUI Mark 3 (HUI3) are based on different subsets of these items (12 items and 13 items, respectively).

^d^Direct communication with the instrument developers.

^e^The SF-6D can be derived from both the 12-item (SF-12) and 36-item (SF-36) Short Form health surveys; the respective descriptive systems comprise 7 of 12 items and 11 of 36 items. The SF-6D is validated only when derived from the larger 12-item or 36-item Short Form health surveys. It is not appropriate to administer only those items that are used in the derivation of SF-6D utility scores and, therefore, participants were asked to consider the SF-36v2 in its entirety.

### Procedure

Each focus group began with the lead investigator describing the objective of the session and, in particular, the purpose of generic HRQoL questionnaires. It was essential that focus group participants were aware that the instruments they were being asked to discuss were designed to be applicable for a broad range of different populations. An experienced qualitative researcher facilitated the focus groups using a structured template. The template, which was seen by the facilitator only, was developed in line with our specific research objectives and included the following broad, open-ended questions: (1) What are your immediate thoughts about questionnaire X? (2) Do you feel that questionnaire X (or particular items within questionnaire X) applies to you? (3) Do you have any further thoughts regarding questionnaire X? (4) Given the objective of these questionnaires, is questionnaire X acceptable to you as a whole? The template included probes for each open-ended question. A copy of the full structured template is available on request. The sequence in which the questionnaires were discussed was varied for each focus group to control for order-effect bias [16]; the respective sequences are reported in Table 1.

### Analysis

Framework analysis was used to analyze the information gathered during the focus group sessions and descriptive statistics were used to characterize the demographics of the sample population. This approach is generally regarded as suitable when there are predetermined issues to explore within a limited time frame [17,18]. The facilitator ensured that unclear statements were clarified immediately during the focus groups and also documented contextual factors or ‘field notes’ (such as focus group disturbances and participant fatigue). This additional information was incorporated into the analysis, where appropriate, to supplement the transcribed data. Each focus group session was digitally recorded for subsequent transcription. Data analysis was conducted using NVivo 9.

The following steps were taken to derive themes from the qualitative data. Firstly, two members of the study team (NS and LE) devised a coding framework after reading independently the focus group transcripts; instances where participants expressed contrasting views about specific issues were also documented. Further discussion within the study team (NS, DGTW and SB) led to refinement of the initial coding framework, reducing the number of identified categories into an appropriate number of coherent themes. Transcripts were re-read (NS and LE) to ensure the final themes provided sufficient coverage of the focus group discussions. Data were then summarized into a matrix table for each outcome measure separately, comprising transcript segments from the focus groups (rows) for each identified theme (columns) [17]. Within these tables the participant number and page and line references were documented to assist with retrieval of original data. The sequence in which the outcome measures were discussed was also noted to provide contextual information during the interpretation phase. Once populated, the content of the matrix tables was examined for patterns and associations by making connections within and between the focus groups for each of the six outcome measures. To aid a better understanding of the associations, the perceived strengths (i.e. positive comments) and perceived limitations (negative comments) were tabulated by theme.

Given the nature of the research question – where the outcome measures are the primary focus of the analysis, not the emerging themes – results are presented by outcome measure. The research was approved by the University of British Columbia Behavioural Research Ethics Board (H12-01138) and Vancouver Coastal Health Authority (Research Study #V12-01138).

## Results

A total of 15 individuals living with SCI participated in one of three focus groups conducted between July and August 2012 (five individuals per focus group). Similar themes emerged across three focus groups (discussed below), indicating saturation. No further sessions were conducted. The final sample included 12 men (80.0%), with an average age of 45.7 years (standard deviation (sd) = 13.8; ranging from 21 to 72). Eight participants had paraplegic injuries (53.3%), seven had tetraplegic injuries (46.7%), and the average length of time since injury was 8.28 years (sd = 4.8).

Five main themes emerged from the framework analysis: general perceptions, comprehensiveness, content, wording, and features. ‘Content’ was separated into subthemes – ‘questions’ and ‘options’ – to reflect distinctions made by the focus group participants. A description of the final coding framework is provided in Table 2. The abbreviations FG1, FG2 and FG3 are used to indicate the respective focus group transcript for each selected quote.

**Table 2:** Details of themes and subthemes comprising the coding framework

**Theme**	**Description**
− **Subtheme**	
Comprehensiveness	Comments regarding the coverage of aspects/issues of quality of life that are appropriate for individuals living with spinal cord injury.
Content	Positive and negative comments made by the participants with respect to the questionnaire *content*.
− Questions	Positive and negative comments made by the participants with respect to the *questions* within the questionnaires.
− Options	Positive and negative comments made by the participants with respect to the *response options* for questions within the questionnaires.
Features	Positive and negative comments made by the participants with respect to the questionnaire *format and layout*.
General perception	Immediate thoughts about the questionnaire. Stand-alone words and statements made by the participants about the questionnaires as a whole.
Wording	Positive and negative comments made by the participants with respect to the *wording* (language/terminology) within the questionnaires.

### 15D

Participants found the variety of questions in the 15D to be good: “… *there were enough different questions that dealt with more specific parameters than lumping everything together*” (FG1). However, in general, the instrument was not considered appropriate for individuals in a wheelchair due, primarily, to the mobility item (item #1): “*I cannot walk without the help of others, you know, but I’m not also completely bedridden and unable to move*” (FG1); “*The first question right there, I didn’t know which one to tick*” (FG3); “*I don’t think mobility is just related to walking*” (FG1). Item #8, which asks about bowel and/or bladder function, also failed to provide respondents with relevant response options: “*I mean I have a slight problem with my bladder whereas I just have to use a catheter. It’s not like I have a real problem with anything*” (FG2); “… *you don’t want to quite say five because that says I have no control of my bladder or bowel functions. I mean you learn to schedule and you make it work*” (FG1). Much of the participants’ dissatisfaction with the 15D was specific to item #1: “*I saw question one and immediately said well this questionnaire has nothing to do with me and skipped it*” (FG2); “… *if it was the last question it probably wouldn’t bother you as much but when you start out a questionnaire like this right off the bat, you know, why am I doing this?*” (FG3).

### AQoL-8D

The general perception of the AQoL-8D was positive in each focus group, with participants commenting on the breadth of the issues covered and the manner in which questions about mobility were incorporated: “*… it asked about pleasure… it did ask about burden, you know, whether you feel you’re a burden, those things are two important questions*” (FG1); “*I like the wording of that one* [item #3]*, it doesn’t exclude anybody getting around, however you get around on your hands or knees or do cartwheels*” (FG3); “… *this is more for spinal cord injury, like how do you feel after your injury with your family and doing activities and stuff*” (FG3). Participants indicated that the 35-item instrument was comprehensive without being burdensome: “*… it’s very in-depth and it totally hit all the marks*” (FG2); “… *it was more satisfying to fill out… I guess that’s maybe one way I would define it, it was after filling it out you felt, okay, good, I was able to, you were at peace. The other ones left you kind of frustrated*” (FG1); “*It took longer but I don’t think it’s the length of time that really bothers us… I think more of it is going through the questionnaire feeling that you’ve adequately expressed yourself*” (FG1).

The response options for AQoL-8D items were also seen as a strength of the instrument: “*I felt I had an option for every one of the 35 questions*” (FG3); “*… question 33, and we’ve raised it a number of times in other questionnaires, which is how often do you feel depressed. And this one gives you enough different categories to better describe the level that you feel*” (FG1). Negative remarks about the AQoL-8D were directed towards item #34, where ‘close,’ ‘intimate’ and ‘sexual’ relationships were combined into a single item: “*… it lumps a lot together, I mean you can have close and intimate relationships that have nothing to do with sex, you know*” (FG1).

### EQ-5D-5L

Comprising only five questions, it was unsurprising that positive statements for the EQ-5D-5L related to brevity, whereas critical comments were concerned with the perceived lack of depth: “*The words are less… it gets right to the point*” (FG1); “*My kind of questionnaire… very simple*” (FG2); “… *too generic, there’s nothing, nothing there*” (FG2); “*… these are five major things but they are small parts of each one*” (FG2). Despite the concision, respondents felt that the instrument had relevance for individuals living with SCI: “*I think this one is more to people with spinal cord injuries*” (FG3); “*This is one that I could fill in and feel comfortable that an individual would have a better idea… some idea about my mobility, my self-care, my usual activities, my pain discomfort, my anxiety, my depression. Yeah this one was done well*” (FG3); “*For me it was good because I fit in there*” (FG1). However, this view was not universal; comments such as, “*… it just doesn’t address quality of life at all… Too brief and too vague*” (FG1) and “*… put that one on the scrap list*” (FG2) were made in relation to the lack of comprehensive coverage provided by the EQ-5D-5L.

The anxiety and depression item was challenged, with participants suggesting they were unable to indicate mood-related impairment using the standardized question and response options: “*I can’t move because I’ve got so much spasms today and there’s nothing I can do about that… I’m feeling down about that but I’m not depressed*” (FG1); “*I’m not a depressed person but I definitely have bad days where my function is not good*” (FG1). The instrument’s recall period (i.e. “your health today”) was viewed positively; “*It’s easier to reply because it’s today*” (FG1).

### Health Utilities Index (HUI)

The HUI was seen as a largely straightforward instrument to complete but there were issues raised regarding the layout: “*… too much like a high school exam*” (FG3); “*For me, there were a lot of words*” (FG1). Although participants commented positively on the variety of issues included in the instrument (“*… what it touches on, it’s a good variety*” (FG2)), many felt that they were unable to accurately describe their quality of life: “*I have a really good quality of life but the way I had to answer the questions didn’t give me an option really to express that*” (FG1); “…*there’s not enough parameters to describe… how you actually deal with pain*” (FG1). Combining multiple concepts into a single question was viewed negatively on multiple occasions. For example, item #13 frames response options around the respondent’s ability to eat, bathe, dress and use the toilet: “*… it lumps together too many parameters*” (FG1). With regard to physical functioning – and item #9 in particular – participants reported that the instrument did not afford wheelchair users the same range of response options as individuals who can walk; “*… question 9, they’re beating around the bush about equipment but they’re not mentioning you’re unable to walk but you are able to get around*” (FG2); “*…* [item #9 needs to be] *defined a little bit more if you’re trying to find wheelchair people*” (FG2).

### Quality of Well-Being Scale Self-Administered (QWB-SA)

The breadth of the QWB-SA was viewed favourably by some individuals when considering the generic nature of outcome measurement: “*I thought it was quite thorough, they asked questions on just about everything that you could possibly go through from sadness to happiness to pain to everything*” (FG3); “*It’s exhaustive in a good sense I think if you are looking to get information for really anything*” (FG2). Negative comments about the comprehensiveness of the instrument related to the lack of relevance for many items: “*… the questions were too vague in scope*” (FG1); “*Well, you know, ninety percent of the time I’m like, ‘Oh yeah, this is an irrelevant question’*” (FG3). Consequently, some individuals failed to see how their responses to the instrument could be useful: “*… I really couldn’t figure out where they were going*” (FG2); “*I have a hard time understanding how this questionnaire can depict me as an individual because it is so broad*” (FG3).

A number of participants thought the questionnaire seemed more like a test than an assessment of health or well-being: “*I was feeling very good and then I felt even better because it seemed like I was passing a test… I think I had some pretty good answers*” (FG3). Positive and negative aspects of the instrument’s layout were also discussed: “*… alternating lines are highlighted so you’re not prone to make, you know, to pick the wrong, the wrong answer*” (FG3); “*Not attractive, it looked messy, unorganized*” (FG1); “*I didn’t want to do it, honestly*” (FG1).

### SF-36v2

Similar to the QWB-SA and HUI, supportive comments for the SF-36v2 reflected participants’ acceptance that generic HRQoL measures are designed to be applicable for a broad range of respondents: “*… this applies to pretty much everybody*” (FG3); “*The questions were, you know, you could answer them with no difficulty at all*” (FG3). The absence of relevance for individuals with SCI was discussed with regard to general perceptions, comprehensiveness and wording: “*It turns me off. I mean there’s no relevancy to me… I’m going to be less inclined to want to do something with that survey*” (FG1); “… *gives me a feeling of things that have nothing to do with me*” (FG3); “… *this one whole section here* [the Physical Functioning subscale]*, I mean none of these apply basically, walking a mile, etcetera.*” (FG3). Questions about mobility raised concern; participants discussed the fact that responses would give a false impression of their health status: “*… the walking one hundred yards, well, I can wheel a hundred yards without even thinking about it, right, but I’m not going to put that on here. I’m going to put no, I can’t do a hundred yards, and they look at this and go oh that’s a problem.*” (FG3); “… *you know, if it said wheeling I can do it no problem, they can gather that my health is pretty good*” (FG3). Others circumvented this problem by reframing the question: “… *when I saw walking, I just kind of took it as wheeling… I’ll just say wheeling instead, I don’t mind crossing that off and putting that so that the person who gets it back sees it*” (FG2).

## Discussion

This qualitative study addressed a narrow research question in relation to the wide-ranging field of economic evaluation, i.e. to what extent do current preference-based HRQoL measures enable individuals living with SCI to *describe* their health state in a manner deemed appropriate to the individuals themselves? Given the paucity of evidence for the empirical validity of any existing instrument these findings provide an important contribution to the literature, allowing for evidence-based consideration of which instruments are suitable candidates for further empirical investigation. In addition, this study provides insight into the potential discrepancies between how the SCI community view their quality of life and how standardized generic instruments permit respondents to describe their quality of life.

A number of key findings emerged. Firstly, given that a primary objective of preference-based HRQoL instruments is to measure health status at an individual level (albeit with health state *values* typically derived at the societal level), it is essential that such instruments have relevance for the intended respondents. For generic measurement of HRQoL, the intended pool of respondents is much broader than a condition-specific outcome measure and, therefore, perceived relevance across a multitude of clinical areas is likely to be a difficult task to achieve for instrument developers. Participants understood the generic objective of the instruments they were asked to discuss as a result of the short explanatory session at the start of each focus group; while a lack of relevance was infrequently viewed with excessive criticism, only the AQoL-8D and EQ-5D-5L were lauded for being applicable to an SCI population. Criticisms relating to content (e.g. irrelevant items or response options, or a general lack of depth) were often framed around participants’ frustration at not being able to adequately describe their health state (15D, EQ-5D-5L, HUI, QWB-SA, SF-36v2). Relating to the perceived objectives of HRQoL instruments, completion of the HUI and QWB-SA was reminiscent of an exam or test. Such observations suggest that respondents do not view these instruments solely as being an attempt to measure quality of life; an exam or test, by definition, can be failed, indicating that there are ‘correct’ answers for some items.

Secondly, our findings indicate that the generic nature of HRQoL questionnaires was not problematic *per se*. As discussed above, perceived relevance of an instrument was the key issue for focus group participants. The main determinant for relevance (and, therefore, general acceptance) was often the item or items that address mobility. All participants expressed positive views toward the AQoL-8D; at some point, the other five questionnaires all refer to walking, whereas the AQoL-8D asks respondents about their ability to ‘get around by yourself’. Interestingly, the mobility item of the EQ-5D-5L (item #1), which frames all five response options around the respondents’ ability to walk (the lowest response option being, ‘I am unable to walk about’) was not discussed in any of the focus groups, whereas any reference to ‘walking’ was a topic of much discussion for the 15D, HUI, QWB-SA and SF-36v2. It is plausible that the concise nature of the EQ-5D-5L, where instructions and words are kept to a minimum, prevents respondents feeling alienated.

A degree of caution is necessary when comparing participants’ discussion of the word ‘walking’ across the instruments. Although no negative comments were specific to the EQ-5D-5L, there was general discussion about how participants interpret the word. While some people take the literal meaning and, accordingly, would provide a response such as ‘I am unable to walk about’ (EQ-5D-5L) or ‘Unable to walk at all’ (HUI), others reframe questions about walking to suit their preferred interpretation of the question (for example, in reference to the SF-36v2, “*I’ll just say wheeling instead, I don’t mind crossing that off and putting that*” (FG2)). These highly divergent approaches are problematic for outcome measurement. Those individuals taking a literal interpretation are likely to provide a markedly different answer to those taking a ‘reframed’ interpretation; these two groups of respondents would provide systematically different responses. The potential for reframing questions has been acknowledged in the SCI literature for some time [19,20], and has led to programs of research that are seeking to identify approaches that improve the applicability of existing quality of life measures for SCI populations [21].

Finally, it was apparent that the length of a questionnaire is not an important factor when respondents are asked to compare outcome measures that measure similar concepts. Although the brevity of EQ-5D-5L was viewed positively by some participants, it was evident that perceived relevance was the most important issue. At 35 items, the third longest survey of those included in the study, the AQoL-8D was not viewed as burdensome by any participant: “*…the length of the questionnaire, it wasn’t, it didn’t put you off because it was a good questionnaire*” (FG1).

### Strengths & limitations

The strengths of the study lay in the selection of instruments, recruitment of participants, and conduct of the focus groups. Working with experienced staff from the Vancouver Spine Program, it was possible to ensure that a range of clinical characteristics were sampled; importantly for the relatively small SCI community, we were able to identify participants without a burdensome recruitment strategy. With regard to conduct, having a focus group facilitator with expertise in qualitative research and data analysis enabled us to maintain consistency across the sessions, while acknowledging the need for participants to have sufficient freedom to discuss issues of importance. Additional steps were taken to address the potential problems of order-effect bias [16], varying the order in which instruments were discussed in the three focus groups. For a preference-based instrument, frequency of use or country of origin was not a barrier to inclusion. The EQ-5D (3-level version) and SF-6D are the most commonly used measures [22], and instruments such as the 15D and AQoL-8D (or other AQoL instruments) are rarely, if ever, used in economic evaluations outside the geographical region where they were developed. These factors do not preclude the possibility that an instrument provides the best system for individuals living with SCI to describe their current health state. As discussed above, it is important to emphasise that this study focuses solely on the descriptive systems of existing instruments. The values attached to the respective finite sets of health states, and the methodologies used to derive such values, are not relevant within the scope of this paper.

Although our findings provide direction for further research, the study and results are not without limitations. Firstly, given the nature of spinal cord injuries (such as differences in the cause of injury, level of injury, and extent of secondary conditions and complications) and the considerable variation in demographics and socio-demographics of those living with SCI (e.g. gender, age, and living conditions), the characteristics and opinions of the 15 participants cannot be considered exhaustive. However, the need for representative samples and the notion of ‘generalizability’ is different in qualitative research compared to quantitative research, where the focus of the former is not to measure, explain, or predict [23,24]. Topic saturation occurred within the conducted focus groups and we believe the emerging themes to be recognisable in the broader SCI context [25].

A second limitation concerns the necessity to restrict the number of preference-based instruments discussed in the focus groups (six questionnaires were selected for the two-hour sessions). It is not feasible to include the descriptive systems of all preference-based instruments in a single comparative study. In addition to the need to limit participant burden and the time constraints inherent in focus group studies, the availability of different versions of similar questionnaires means that choices had to be made. For example, a suite of AQoL questionnaires is available, including 4-item, 6-item, 7-item and 8-item variants [26]. Similarly, the EuroQol Group have 3-level and 5-level instruments [27], and the SF-6D descriptive system differs depending on whether it is derived from the SF-12 or the SF-36 [15,28]. The issue is further complicated with respect to the SF-6D because the measure is validated only when derived from the larger 12-item or 36-item Short Form health surveys; it is not appropriate to administer only those items that are used in the derivation of SF-6D utility scores. Where necessary, we selected one descriptive system per ‘family’ (i.e. one of the AQoL instruments, one of the EuroQol Group instruments, etc.), choosing the instrument with the highest number of items each time. Finally, whether or not the varied sequencing of discussion for the six instruments eliminated order-effect bias is impossible to answer in a qualitative study and, therefore, should be highlighted as a potential limitation. Comments for the QWB-SA were positive only when it was the first instrument discussed (FG3). Similarly, the SF-36v2 was viewed negatively by all participants when it was discussed last (FG1). It is important to note that the AQoL-8D was the preferred instrument in all three focus groups.

## Conclusions

With regard to the descriptive systems of existing preference-based HRQoL instruments, our qualitative findings suggest that individuals living with SCI identified a ‘favourite’ (AQoL-8D), potential options (EQ-5D-5L, HUI and SF-36v2) and lesser preferred alternatives (QWB-SA and, in particular, 15D). There are many pragmatic considerations to take into account when selecting a measure of health benefit for the purposes of economic evaluation. For decision makers, enabling comparability of study findings both within and across clinical areas is a key requirement, meaning that the relatively underused suite of AQoL instruments is at a significant disadvantage compared to more established measures. However, the world of preference-based measures is not a closed shop and further research is a necessary pursuit if evidence-based improvements are to be made to the current methodological toolkit used in economic evaluation [29]. Empirical, comparative analysis of the favourable instruments identified in this qualitative study is a much-needed starting point within the context of spinal cord injury.

## Abbreviations

AQoL: Assessment of Quality of Life; AQoL-8D: Assessment of Quality of Life 8-dimension questionnaire; HRQoL: Health-related quality of life; HUI: Health Utilities Index; HUI2: Health Utilities Index Mark 2; HUI3: Health Utilities Index Mark 3; QALY: Quality-adjusted life year; QWB-SA: Quality of Well-Being Scale Self-Administered; SCI: Spinal cord injury; SF-12: Medical Outcomes Study 12-item Short Form Health Survey; SF-36: Medical Outcomes Study 36-item Short Form Health Survey; SF-36v2: Medical Outcomes Study 36-item Short Form Health Survey (version 2).

## Competing interests

The authors declare that they have no financial or non-financial competing interests.

## Authors’ contributions

DGTW, NM, VKN, MFSD and SB were involved in the conception and design of the study. NS facilitated the focus groups, with assistance from DGTW and LE. NS and LE performed data analysis. DGTW drafted the original manuscript and was responsible for the final submission. All authors were involved in the review of draft manuscripts and read and approved a final version prior to submission.

## Authors’ information

This project is an integral part of a program of research exploring the use of preference-based health-related quality of life measures in the context of spinal cord injury. This research program has brought together a team comprising academics and clinicians from the fields of health economics, qualitative research, rehabilitation science and spinal cord injury.
